# Recognizing Indigenous peoples’ and local communities’ rights and agency in the post-2020 Biodiversity Agenda

**DOI:** 10.1007/s13280-021-01561-7

**Published:** 2021-05-18

**Authors:** Victoria Reyes-García, Álvaro Fernández-Llamazares, Yildiz Aumeeruddy-Thomas, Petra Benyei, Rainer W. Bussmann, Sara K. Diamond, David García-del-Amo, Sara Guadilla-Sáez, Natalia Hanazaki, Nicolas Kosoy, Margarita Lavides, Ana C. Luz, Pamela McElwee, Vicky J. Meretsky, Teresa Newberry, Zsolt Molnár, Isabel Ruiz-Mallén, Matthieu Salpeteur, Felice S. Wyndham, Francisco Zorondo-Rodriguez, Eduardo S. Brondizio

**Affiliations:** 1 Institució Catalana de Recerca i Estudis Avançats (ICREA), Barcelona, Spain; 2 Institut de Ciència i Tecnologia Ambientals (ICTA), Universitat Autònoma de Barcelona (UAB), Carrer de les columnes, s/n. Z-building (ICTA-ICP), Bellaterra Campus, Cerdanyola del Valles, Bellatera, 08193 Barcelona, Spain; 3 Helsinki Institute of Sustainability Science (HELSUS), Faculty of Biological and Environmental Sciences, University of Helsinki, P.O. Box 65, (Viikinkaari 1), 00014 Helsinki, Finland; 4 Centre for Functional and Evolutionary Ecology, University Montpellier, CNRS, CEFE, UMR 5175, 1919, Route de Mende, 34293 Montpellier, France; 5 Department of Ethnobotany, Institute of Botany and Bakuriani Alpine Botanical Garden, Ilia State University, Tbilisi, Georgia; 6 Department of Geography and the Environment, University of Texas, Austin, USA; 7 College of Liberal Arts, The University of Texas at Austin, 116 Inner Campus Dr. Stop G6000, Austin, TX 78712 USA; 8 Independent Researcher, Barcelona, Spain; 9 Departamento de Ecologia e Zoologia, Universidade Federal de Santa Catarina, ECZ/CCB/UFSC, Campus Trindade s/n, Florianópolis, SC 88010-970 Brazil; 10 Faculty of Agricultural and Environmental Sciences, McGill School of Environment, McGill University, Macdonald Stewart Building, MS3-037, Macdonald Campus, Ste. Anne-de-Bellevue, Quebec, H9X 3V9 Canada; 11 Earth Law Center, New York, NY USA; 12 ISEG- Lisbon School of Economics & Management, Universidade de Lisboa, Lisbon, Portugal; 13 Department of Human Ecology, Rutgers University, 55 Dudley Road, New Brunswick, NJ 08901 USA; 14 O’Neill School of Public and Environmental Affairs, Indiana University, Bloomington, IN 47405 USA; 15 Department of Science, Tohono O’odham Community College, Sells, 1830 E. Broadway, Ste 124-202, Tucson, AZ 85719 USA; 16 Centre for Ecological Research, ELKH, Alkotmány u. 2-4, Vácrátót, 2163 Hungary; 17 Internet Interdisciplinary Institute, Universitat Oberta de Catalunya, Av. Friedrich Gauss, 5, Castelldefels, 08860 Barcelona, Spain; 18 Patrimoines Locaux, Environnement et Globalisation (UMR 208 PALOC), IRD, MNHN, French National Research Institute for Sustainable Development (IRD), 57 rue Cuvier, CP 51, 75231 Paris Cedex 05, France; 19 School of Anthropology and Museum Ethnography, University of Oxford, Oxford, UK; 20 PO Box 3162, Santa Cruz, CA 95063 USA; 21 Departamento de Gestión Agraria, Facultad Tecnológica, Universidad de Santiago de Chile, Santiago, Chile; 22 Department of Anthropology, Indiana University Bloomington, 702 E. Kirkwood Ave. Student building 130, Bloomington, IN 47401 USA

**Keywords:** Biodiversity policy, Convention on Biological Diversity, Indigenous and local knowledge, Nature’s values, Right-based approach

## Abstract

The Convention on Biological Diversity is defining the goals that will frame future global biodiversity policy in a context of rapid biodiversity decline and under pressure to make transformative change. Drawing on the work of Indigenous and non-Indigenous scholars, we argue that transformative change requires the foregrounding of Indigenous peoples’ and local communities’ rights and agency in biodiversity policy. We support this argument with four key points. First, Indigenous peoples and local communities hold knowledge essential for setting realistic and effective biodiversity targets that simultaneously improve local livelihoods. Second, Indigenous peoples’ conceptualizations of nature sustain and manifest CBD’s 2050 vision of “Living in harmony with nature.” Third, Indigenous peoples’ and local communities’ participation in biodiversity policy contributes to the recognition of human and Indigenous peoples’ rights. And fourth, engagement in biodiversity policy is essential for Indigenous peoples and local communities to be able to exercise their recognized rights to territories and resources.

## Introduction

The Convention on Biological Diversity (CBD) is now working to formulate the goals that will frame global biodiversity policy for decades to come. The Parties to the Convention are doing so while facing the fact that they failed to achieve most of the targets of the 2011–2020 Strategic Plan for Biodiversity while global biodiversity continues to decline precipitously (Green et al. 2019). Moreover, the window of opportunity to take action is narrowing (Díaz et al. 2019; IPBES 2019a, b). To slow biodiversity loss, transformative change, i.e., a fundamental system-wide reorganization, is needed in the ways biodiversity policies are designed, implemented, and enforced, from international to national scales, and across sectors (Díaz et al. 2020).

In this ‘Perspective’, we argue that transformative change requires the foregrounding of Indigenous peoples and local communities’ (IPLC) rights and agency in biodiversity policy. Much of the world’s biodiversity now exists in landscapes and seascapes traditionally owned, managed, used and/or occupied by IPLC (Brondizio and Le Tourneau 2016; Garnett et al. 2018). Moreover, despite increasing resource extraction pressures (Díaz et al. 2019) and growing violence against IPLC who are defending their territories and resources (Scheidel et al. 2020), biodiversity is declining more slowly in areas managed by IPLC than elsewhere (Garnett et al. 2018; Fa et al. 2020; O’Bryan et al. 2020).

However, IPLC continue to face challenges to full participation in the crafting and implementation of biodiversity policy at local, regional, and global levels (Witter et al. 2015; Forest Peoples Programme et al. 2020). For example, while  about 40% of terrestrial protected areas overlap with IPLC lands (Garnett et al. 2018), IPLC only formally govern < 1% of them (UNEP-WMCM et al. 2018). Further, the current zero draft of the post-2020 biodiversity framework continues to make the same long-standing calls for promotion of traditional knowledge and “full and effective participation” of IPLCs without the more concrete measures they have requested (see Box).

Four key points underscore the importance of recognizing IPLC rights and agency in biodiversity policy. First, IPLC hold knowledge essential to setting realistic and effective biodiversity targets that simultaneously improve local livelihoods. Second, IPLC conceptualizations and understandings of nature are aligned with CBD's 2050 vision. Third, IPLC participation in biodiversity policy as rights-holders enhances the social elements needed to meet CBD 2050 vision. And fourth, engagement in biodiversity policy is essential for Indigenous peoples and local communities to be able to fully exercise the rights to territories and resources that have been recognized by international agreements. We draw on the work of both Indigenous and non-Indigenous scholars to illustrate these four key points. We acknowledge that we are non-Indigenous academics working in partnership with and informed by IPLC and their representatives. We do not claim to speak on behalf of IPLC, but we gratefully acknowledge the depth of knowledge and perspectives shared with us over the years that shape this ‘Perspective’.

BOX: IPLC contributions to the post-2020 framework Preliminary discussions for the post-2020 global biodiversity framework clearly indicated that it “should effectively incorporate gender considerations and the perspectives of Indigenous peoples and local communities” (CBD 2019). Consultations were held with IPLCs to develop the framework in two Global Thematic Dialogue for Indigenous Peoples and Local Communities in 2019–2020, as well as through 17 preparatory webinars across seven regions organized by the International Indigenous Forum on Biodiversity (IIFB) to gather inputs. Other Indigenous-led organizations like the Asia Indigenous Peoples Pact and the Consejo Indígena de Mesoamérica, as well as groups that work closely with IPLCs, like the Indigenous and Community Conserved Areas (ICCA) Consortium, also submitted formal written recommendations to the process. These organizations emphasized the need for the framework to explicitly recognize territorial rights for IPLCs, to request protection for threatened land defenders, and to address mutual sustainability of humans and biodiversity (ICCA 2018; IIFB 2019). For example, one key demand was that target 1 “must include the legal recognition and protection for the territories, land and water of Indigenous peoples and local communities” (CBD 2021). Yet these recommendations are not reflected in the current text of the zero draft (as of early 2021), with only Target 20 addressing IPLCs: “By 2030, ensure equitable participation in decision-making related to biodiversity and ensure rights over relevant resources of Indigenous peoples and local communities, women and girls as well as youth, in accordance with national circumstances.” This weaker approach prompted Indigenous participants in the second Global Thematic Dialogue to write to CBD that “the contributions, values, perspectives, knowledge and text proposals of Indigenous peoples and local communities were not adequately taken into account in the updated zero draft of the global biodiversity framework” (CBD 2021).

## IPLC hold knowledge essential to setting realistic and effective biodiversity targets that simultaneously improve local livelihoods

Scholars and practitioners acknowledge the importance of IPLC’s knowledge in advancing scientific understanding of nature (e.g., Athayde et al. 2017; Joa et al. 2018; see also Gadgil et al. 1993; Berkes et al. 2000; Gadgil et al. 2021 for seminal papers on the topic). Indigenous and local knowledge (ILK) has advanced scientific understandings of species’ ranges, baselines, and trends and contributed to mapping, monitoring, and reporting changes in local biodiversity, including collective evidence of resource over-exploitation, invasive species expansion, pollution, and climate-change impacts (e.g., IPBES 2019a, b; Forest Peoples Programme et al. 2020). As ILK contributions are more valuable when knowledge is embedded into sociocultural contexts, there have been a number of initiatives led by Indigenous peoples and local community organizations to enact a range of monitoring activities on the health of biodiversity, climate-change impacts, effects of unsustainable activities, or implementation of international agreements such as the CBD (Farhan Ferrari et al. 2015; Tengö et al. 2017). For example, the *Local Biodiversity Outlook 2,* a report compiled in partnership with IPLCs and issued to complement the *Global Biodiversity Outlook 5*, provides many examples of such on-the-ground initiatives and their contributions to the implementation of the Strategic Plan for Biodiversity 2011–2020 (see Forest Peoples Programme et al. 2020). Nevertheless, current efforts to design and implement a post-2020 global biodiversity framework do not fully acknowledge the importance of ILK for better stewardship of our planet (see Box). For example, while at least 36% of intact forest landscapes are within Indigenous people’s lands (Fa et al. 2020), efforts to preserve these landscapes generally do not recognize the multiple values of ILK in their management (Zanotti and Knowles 2020).

Moreover, the holistic nature of ILK is also essential in CBD’s quest to set interdependent and mutually reinforcing targets and to minimize trade-offs among targets, including targets set by other international agreements including the Sustainable Development Goals or the United Nations Declaration on the Rights of Indigenous Peoples. For example, protected area expansion can trade-off against IPLC’s livelihood options, quality of life, and rights (e.g., Agrawal and Redford 2009; Sayer et al. 2021). Ignoring IPLC and their knowledge in decisions regarding protected area establishment and management would likely be disruptive for local communities and result in conservation failures (Whyte 2018), reinforcing sentiments that conservation can be a colonial enterprise (Whyte 2017). Alternatively, the co-production of new knowledge based on evidence from both science and ILK could contribute to setting realistic and effective biodiversity targets that simultaneously improve local livelihoods (Tengö et al. 2014). Examples exist in which partnership between researchers and IPLC have resulted in emergent knowledge that supports conservation, including for culturally important species and ecosystems (Sterling et al. 2017), and social aspects, such as local governance (Beveridge et al. 2020).

Lack of IPLC agency in biodiversity policy runs the risk of using ILK only for utilitarian ends, particularly if ILK is decontextualized, co-opted and/or instrumentalized for conservationists’ purposes, rather than understood as a relational expression based on human-to-nature appreciation and responsibilities (Whyte 2013; Todd 2016). Recognizing IPLC rights and agency in biodiversity management and policy can help prevent conflicts that might arise from extractive uses of ILK (Bohensky and Maru 2011). Transformative change requires that biodiversity policy recognize different worldviews and local forms of relationship to nature, including the emphasis that ILK systems place on nurturing responsible relationships among humans and non-humans (McGregor et al. 2018). Such change also requires that biodiversity policy safeguards IPLC knowledge sovereignty (Kukutai and Taylor 2016), provides IPLC with better access to scientific traditions as a resource for their own purposes, and ultimately accommodates diverse knowledge systems (Díaz-Reviriego et al. 2019).

## IPLC understandings of nature are aligned with the CBD’s 2050 vision of “*Living in harmony with nature*”

Worldviews that separate humans from the ecosystems they rely on are recognized as an indirect cause of biodiversity decline (Díaz et al. 2019; Forest Peoples Programme et al. 2020). In contrast, IPLC often understand nature as an interconnected web of life, linking humans and non-humans in complex relations (e.g., Lyver et al. 2017; Reo 2019). In such conceptualizations, humans are viewed as an integral component of nature (Coscieme et al. 2020) and nature is imbued with social, cultural and spiritual values (Berkes 2017). Moreover, IPLC conceptualizations of nature often draw on stewardship ethics based on mutual reciprocity between humans and nature, temporary custody for future generations, and health of and attachment to land (Pascua et al. 2017; Reo 2019). These conceptualizations are also dynamic and adapt to external environmental changes (McMillen et al. 2017). They form the basis for land and seascape management, including the protection of sacred areas or species, taboo enforcement, or selective cutting and burning (e.g., Joa et al. 2018). For example, sacred forests -where no extractive activities occur- are common in IPLC lands and allow for the maintenance of forest cover and structure (Samakov and Berkes 2017). Many IPLC also limit the exploitation of resources for certain periods of time or seasons to ensure the maintenance and natural recovery of ecosystems, including forest areas, natural pastures or river sections (e.g., Hammi et al. 2010). Many examples show that management by customary institutions results in more sustainable and productive systems, in ecosystem restoration, or in combatting pollution (e.g., Hoover et al. 2012; Ens et al. 2016; Reyes-García et al. 2019; Fernández-Llamazares et al. 2020).

The acknowledgement and inclusion of IPLC’s understandings of nature in biodiversity policy design and implementation can be vital to set goals to achieve the CBD’s 2050 vision (Sterling et al. 2017). For example, IPLC’s conceptualizations of nature can extend ethical concerns to diverse species and natural elements by giving more importance to relational values of nature (e.g., Pascua et al. 2017; Reo 2019). Indeed, examples exist in which IPLC values recognizing the rights of ecosystems to exist, reproduce, and thrive have been enshrined in policy instruments, such as the Ecuadorian Constitution and the Bolivian law in which Mother Earth is granted rights and New Zealand’s recognition of the legal personhood of the Whanganui River (Chapron et al. 2019).

Failure to recognize IPLC’s rights and agency in designing and implementing biodiversity policy disregards the existence of these different ways to relate with nature. For some authors, this transforms environmental management into a transactional enterprise in which nature is considered a “resource”, and the imposition of a single conservation model leads to the erosion of alternative worldviews (e.g., Nadasdy 2003; Eichler and Baumeister 2018). Transformative change requires heeding IPLC voices in global discussions on the collective future of humanity and the planet (McGregor et al. 2020) and extending current biodiversity policy frameworks to accommodate IPLC understandings of nature (Tengö et al. 2014; Lyver et al. 2016).

## IPLC participation in biodiversity policy as rights-holders enhances the social bases needed to meet CBD’s 2050 vision

IPLC are politically active in the defense of biodiversity, ranging from participating in different scales of governance to resisting environmentally degrading activities. At the local level, lands managed by IPLC have better nature conservation outcomes than other areas (e.g., Garnett et al. 2018; Tran et al. 2020). Moreover, many IPLC participate in monitoring systems to inform implementation and development of conservation indicators and actions (Ens et al. 2016). At the global level, IPLC are increasingly active and insisting on participating in environmental negotiations and intergovernmental processes, such as the involvement of the International Indigenous Peoples’ Forum on Climate Change in the 2015 Paris climate conference, where they succeeded in drawing attention to their presence and claims for rights through creative spaces and events (Suiseeya and Zanotti 2019). In other examples, the Inuit Circumpolar Council has been very active in international policy development to reduce pollution burdens, helping shape the Stockholm Convention on Persistent Organic Pollutants, or the Minamata Convention on Mercury (see Fernández-Llamazares et al. 2020). Similarly, Indigenous peoples’ organizations such as the Coordinator of Indigenous Organizations of the Amazon River Basin (COICA, for its Spanish acronym) or Tebtebba (Indigenous Peoples’ International Center for Policy Research and Education) have played crucial roles in the recognition of Indigenous peoples’ rights in the context of UN REDD + negotiations (e.g., Walbott 2014). Using methods that range from direct action to legal claims against transnational corporations, IPLC have also been active political actors against activities potentially leading to environmental degradation including mining operations, hydrocarbon exploration, infrastructure development, and toxic waste dumping (e.g., Spice 2018; Kuokkanen 2019).

A lack of recognition of IPLC rights and their equal participation as stakeholders and leaders in designing biodiversity policy can alienate IPLC by controlling and regulating their resources through processes and institutions that may conflict with their worldviews (Richmond et al. 2013; Tauli-Corpuz et al. 2018). This non-inclusive environmental governance results in IPLC’s resistance and non-compliance with top-down environmental regulations that generate conflict over land and resources, forced displacements, and human suffering (Tauli-Corpuz et al. 2018).

Transformative change that includes IPLC’s agency in biodiversity policy requires recognizing IPLC stewardship and their effective role in sustaining nature (Armstrong and Brown 2019), promoting IPLC decision-making rights, respecting IPLC laws, principles, and customary practices, and addressing Indigenous peoples’ relations with states (Whyte 2017). Transformative change also requires accounting for the negative impacts of agricultural activities, resource extraction, or infrastructural development on nature and for the rights of IPLC (and society in general) to resist such nature damaging activities (IPBES 2019a, b).

## Engagement in biodiversity policy is essential for Indigenous peoples to be able to exercise their recognized rights to territories and resources

The Universal Declaration of Human Rights enshrines fundamental human rights to life, liberty, and security, while the United Nations Declaration on the Rights of Indigenous Peoples recognizes their right to self-determination, territories, and resources. Despite these legal recognitions, and compared to other populations, Indigenous peoples often suffer disproportionately from violations of their tenure, access, and resource rights (Giunta 2019; Forest Peoples Programme et al. 2020). For example, resources that support IPLC livelihoods and spiritual and cultural needs are threatened by extractive industries, intensive agriculture, unsustainable fishing, environmental pollution and spread of invasive species (e.g., Hoover et al. 2012; FAO 2016; Kuokkanen 2019). These activities often result in loss of livelihoods, multiple insecurities, land conflicts, and even violence in the name of vested economic or government interests (Martinez-Alier et al. 2016; Scheidel et al. 2020).

When IPLC are not included as equal stakeholders and leaders in biodiversity policy design and implementation, at national and international levels, their power to exercise and defend their recognized rights, including land tenure, resource and access rights, is diminished. For example, setting a target of protecting 30% of the Earth without IPLC’s involvement would not only disempower them, but the implementation of the target might result in numerous violations of their rights. Transformative change in how biodiversity policies are designed and implemented requires not only addressing IPLC’s territorial concerns (Zurba et al. 2019) and respecting their governance institutions and practices (Artelle et al. 2019), but also including them as rights-holders in the design and implementation of biodiversity policy at multiple levels.

## Conclusion

IPLC contributions to the enhancement and maintenance of biodiversity in land and seascapes are increasingly acknowledged and a growing number of strategies are being mobilized to work with IPLC in conservation policy (e.g., Hill et al. 2020; McElwee et al. 2020). Yet in the zero draft of the post-2020 global biodiversity framework there is little innovation regarding IPLC rights and agency. Transformative change will require recognizing IPLC rights and agency in biodiversity policy, requiring diverse mechanisms that range from fully acknowledging IPLC contributions and the potential social impacts of conservation, to generating equitable and constructive spaces for dialogue, representation in formal decision-making bodies, state acknowledgement of territorial rights, and developing financial mechanisms that allow engagement and leadership in biodiversity policy design and implementation (Forest Peoples Programme et al. 2020).

Nature’s decline is the result of direct and indirect drivers of change—including demographic, economic, political, and institutional arrangements—underpinned by societal values (Díaz et al. 2019). We acknowledge that these drivers interact with one another to impact nature and that the point addressed here is only a piece of the complex puzzle leading to nature’s decline. However, we defend that to accomplish the CBD’s 2050 vision for biodiversity, global biodiversity institutions, supported by member states, should fully embrace and embody the role of IPLC in the transformative change so widely called upon.
